# Novel Metrics to Characterize In Vitro Pollen Tube Growth Performance of Apple Cultivars

**DOI:** 10.3390/plants10071460

**Published:** 2021-07-16

**Authors:** Stefan Roeder, Sara Serra, Stefano Musacchi

**Affiliations:** 1 Tree Fruit Research and Extension Center, Department of Horticulture, Washington State University, Wenatchee, WA 98801, USA; stefan.roeder@wsu.edu (S.R.); sara.serra@wsu.edu (S.S.); 2 Department of Horticulture, Washington State University, Pullman, WA 99164, USA

**Keywords:** pollen viability, *Malus domestica* (Borkh.), in vitro pollen germination assay, categorization

## Abstract

In vitro germination assays are frequently used in screening trials to evaluate the pollen viability of pollinizers. To be effective, screening trials must have defined threshold criteria, from which individuals can then be assessed. However, despite decades of research on pollen viability, no established threshold is available to categorize apple cultivars based on their in vitro pollen tube lengths. This study aimed to identify and characterize the subgroups of cultivars based on their pollen tube growth performance. In vitro pollen tube lengths of 41 individuals were determined by incubating samples on artificial germination media at 15 and 25 °C. A six-number summary statistic was calculated, and hierarchical clustering on principal component (HCPC) analysis was used to determine and characterize subgroups. Furthermore, a decision tree model was used to predict class membership for future datasets. HCPC analysis partitioned the 41 individuals into three subgroups with different performances. The decision tree quickly predicted the cluster membership based on the second quartile at 15 °C and the third quartile at 25 °C. The thresholds from the decision tree can be used to characterize new observations. The use of the methods will be demonstrated using a case study with 29 apple accessions.

## 1. Introduction

Apple (*Malus domestica* Borkh.), like many other tree fruit species, can require cross-pollination with compatible pollen to overcome self- and cross-incompatibility and achieve an economically acceptable yield [1,2]. Standard practice for single cultivar plantings includes pollinizer trees to provide pollen that will be transported using pollinators such as honey, bumble, or blue orchard bees [3,4,5]. Pollinizers are specifically selected for their *S*-genotype, flowering time and duration, and pollen viability [6]. All characteristics are equally important because they all can significantly affect fertilization.

Pollen viability can be evaluated using different in vitro or in vivo protocols, where in vitro approaches usually precede in vivo experiments in screening trials. Various in vitro stain methods can be used to assess pollen viability and germinability. Three of the most frequently utilized metrics are the viability rate, based on fluorescent or non-fluorescent stains, the germination rate, and pollen tube length. In general, viability stains are considered to be faster and easier alternatives to germination tests [7]. In fact, viability stains do not have to be optimized to meet certain genus or species-specific requirements [8] and are less sensitive to environmental effects [9]. However, several reports have indicated a low correlation between pollen viability and germination rates [7,8]. In some cases, traditional staining techniques, such as Alexander’s stain [8], Baker’s procedure [10], iodine potassium iodide [11], and X-Gal [10] produced unreliable results because the staining technique can induce false-positive results by identifying unviable pollen grains as viable.

In vitro germination assays have been used since the early 1900s to investigate a broad set of research questions regarding pollen viability and performance. Since then, several research groups have focused on optimizing certain conditions, such as incubation temperature [12,13], germination media [12,14], and storage conditions [15,16]. There are currently over 35 published germination media for apple pollen that contain different amounts of sucrose, boric acid, calcium nitrate, magnesium sulfate, and potassium nitrate. In addition, different studies utilized various germination techniques, such as hanging or sitting drops [12], agar plates [17], or cellulose membranes [18].

A further challenge of in vitro viability and germination studies is the lack of standardization among methods using established thresholds or standard cultivars with a known performance that can be used as internal controls across all experimental studies. Thus, different studies have used various arbitrary benchmarks to characterize pollen viability. Florin [19], for example, rated various apple and pear cultivars based on their pollen germination rate as poor (<30%), average (30–70%), or good (>70%) pollen producer. Screening and evaluating a large set of individuals can be a further challenging aspect for researchers, especially if multivariate datasets must be analyzed to determine the cultivar’s performance. Conventional methods are often based on analysis of variance (ANOVA) or non-parametric equivalents (e.g., Kruskal-Wallis H test), followed by multiple comparison procedures such as Dunnett (one vs. all), Tukey (all vs. all), or Games-Howell (all vs. all, non-parametric) tests. A typical problem with these statistical testing procedures, except for multivariate ANOVA and the multiple comparison problem, is their singular focus on one variable. Alternatives used to characterize individuals include unsupervised techniques such as principal components analysis (PCA) or one of many clustering algorithms.

In contrast to supervised methods, unsupervised techniques do not require any classification of the individuals, making it a suitable tool to evaluate individuals with an unknown performance. PCA has previously been used in horticultural screening applications among cultivars or species. Kakani et al. [20] evaluated the heat tolerance of 21 groundnut individuals and 16 cotton cultivars [21]. Sorkheh et al. [22] discriminated eight almond species based on their tolerance to saline conditions, and Ranasinghe et al. [23] determined high temperatures tolerance of eight coconut hybrids. Akšić et al. [24] identified the essential biological reproductive variables and classified 41 sour cherry genotypes based on their reproductive biology.

Clustering is another unsupervised method utilized in horticulture. It aims to group observations based on their similarity. In general, clustering includes different types of algorithms, such as partitioning methods (e.g., K-means, K-medoids), hierarchical (bottom-up, top-down approach), fuzzy, density, and grid- or model-based clustering [25]. Each algorithm’s performance depends on certain parameters that can be modified [26]. K-means and hierarchical clustering, for example, have been used in horticulture to classify the fire blight resistance of 94 apple cultivars [27]. Sochor et al. [28] classified 239 apricot cultivars based on their amino acid content, while Banerjee et al. [29] characterized 10 rice cultivars based on their yield. Gregorio et al. [30] differentiated between two cherimoya cultivars picked at different harvest times based on fruit quality traits. Both PCA and clustering algorithms combined with predictive modeling could be useful tools for screening larger sets of pollinizers and improving comparability across different studies. Thus, this study’s main objectives are threefold: (1) to propose a standardized in vitro protocol to determine the pollen tube performance of apple pollen; (2) to identify and characterize meaningful subgroups using hierarchical clustering on principal components; and (3) to demonstrate the use of predicted projections on a case study with 29 new apple accessions to characterize their pollen tube growth performances.

## 2. Results

### 2.1. Effects of Incubation Time, Germination Media, and Techniques on Pollen Tube Growth

#### 2.1.1. Effect of Incubation Time

**Figure 1:**
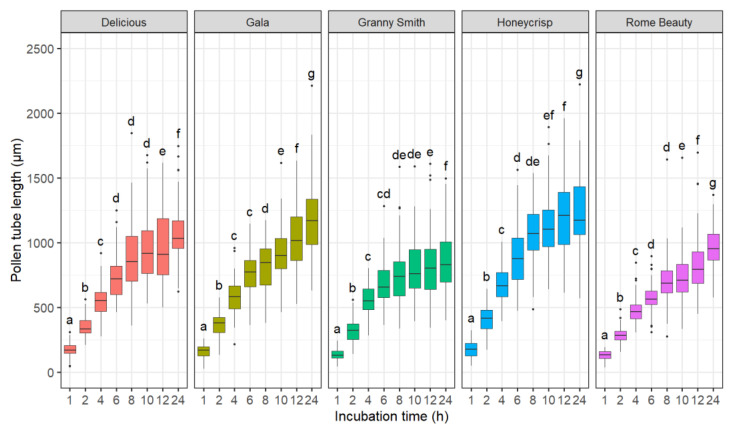
Effect of incubation time on in vitro pollen tube length of ‘Delicious’, ‘Gala’, ‘Granny Smith’, ‘Honeycrisp’, and ‘Rome Beauty’. Different letters indicate significant differences among incubation times within cultivars based on Games-Howell test (*p* < 0.05).

The main effects of incubation time (*p* < 0.001), cultivar (*p* < 0.001), and temperature × cultivar interaction term (*p* < 0.001) were significant. Consequently, analyses were performed separately for every cultivar. The incubation time influenced the pollen tube length of all five cultivars (Figure 1). ‘Gala’, ‘Delicious’, and ‘Rome Beauty’ differed between the 12- and 24-h incubation times, indicating that the maximum pollen tube length was not reached after 12 h. In all cultivars, the average pollen tube growth rate peaked 2 h after plating the suspension and ranged between 142 ± 28 µm h^−1^ in ‘Rome Beauty’ and 206 ± 47 µm h^−1^ in ‘Honeycrisp’. The slowest growth rates were observed 24 h after plating and were 36 ± 9, 40 ± 7, 45 ± 9, 49 ± 12, and 52 ± 13 µm h^−1^ in ‘Granny Smith’, ‘Rome Beauty’, ‘Delicious’, ‘Gala’, and ‘Honeycrisp’, respectively.

#### 2.1.2. Effect of Germination Medium

**Figure 2:**
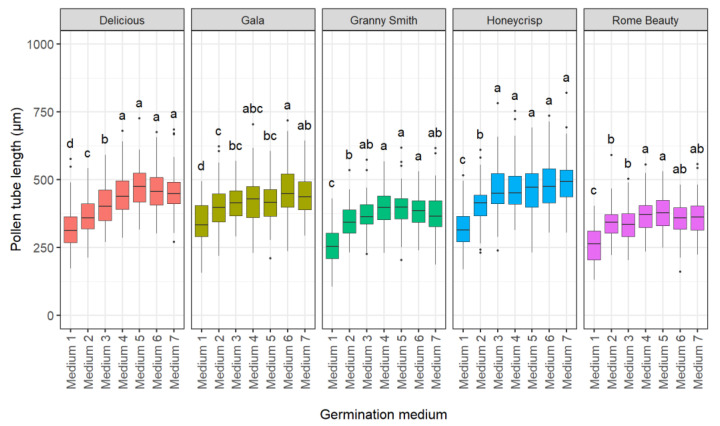
Effect of germination media on in vitro pollen tube length of ‘Delicious’, ‘Gala’, ‘Granny Smith’, ‘Honeycrisp’, and ‘Rome Beauty’. Different letters indicate significant differences among germination media within cultivars based on Fischer’s LSD test (*p* < 0.05).

Six different germination media were compared to an untreated control medium (deionized water) and the standard medium (current publication; Figure 2). Germination medium (*p* < 0.001), cultivar (*p* < 0.001), and medium x cultivar (*p* < 0.001) interactions were significant. Medium 1 (deionized water) resulted in the shortest pollen tube. Six germination media outperformed the control (medium 1). Medium ID 2 published by Calzoni et al. [12] resulted in a shorter pollen tube length in ‘Honeycrisp’ and ‘Delicious’ compared to the other five germination media. The four media containing calcium nitrate (media ID 2, 4, 5 and 6) were not superior to the two media without calcium nitrate (media ID 2 and 7).

#### 2.1.3. Effect of Germination Technique

**Figure 3:**
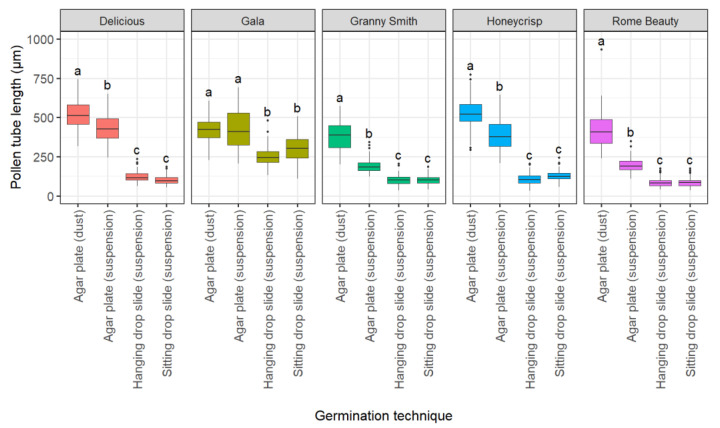
Effect of germination technique on in vitro pollen tube length of ‘Delicious’, ‘Gala’, ‘Granny Smith’, ‘Honeycrisp’, and ‘Rome Beauty’. Different letters indicate significant differences among germination techniques within cultivars based on Fischer’s LSD test (*p* < 0.05).

The germination technique considerably affected pollen tube length (Figure 3). Germination system (*p* < 0.001), cultivar (*p* < 0.001), and germination technique × cultivar interaction (*p* < 0.001) terms were significant. The dusting of pollen onto agar plates resulted in the highest average pollen tube lengths. In contrast, the use of the hanging (mean = 134 µm, sd = 72 µm) and sitting drop (mean = 143 µm, sd = 93 µm) methods resulted in the shortest pollen tubes. Pipetting pollen suspensions on agar plates resulted in longer pollen tubes in all five cultivars (mean = 326 µm, sd = 138 µm) than the two drop techniques. However, the pollen tube length was still short compared with the dusting method (mean = 454 µm, sd = 108 µm). Overall, the average pollen tube length was reduced by 28% in the agar plate (suspension) method, 71% in the hanging drop method, and 68% in the sitting drop method compared to the agar plate (dusting) method.

### 2.2. Identification and Characterization of Subgroups

#### 2.2.1. Principal Component Analysis (PCA)

**Figure 4:**
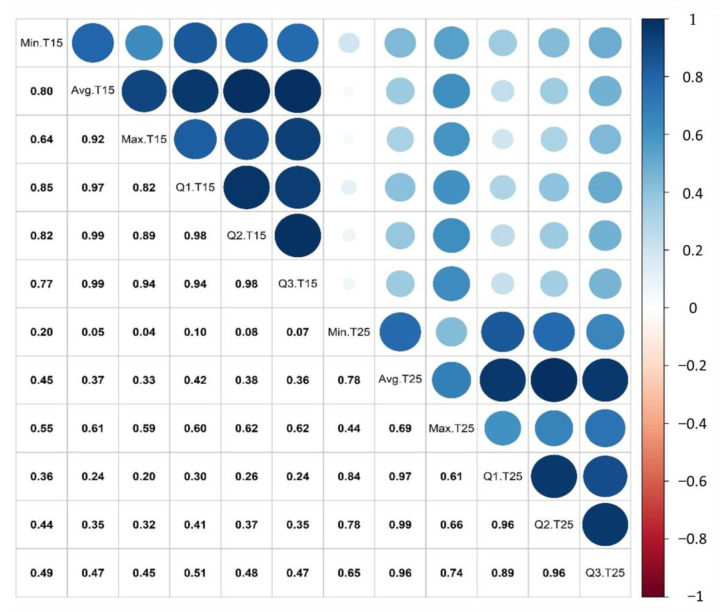
Spearman correlation coefficients between variables. Variables include the minimum (Min.), average (Avg.), maximum (Max.), first (Q1), second (Q2) and third quantile (Q3) of the pollen tube length measured at 15 °C (T15) and 25 °C (T25). Data were combined from 41 individuals. Color bar on the right represents the range of the correlation coefficient, ranging from −1 (dark red, strong negative correlation) to 1 (dark blue, strong positive correlation).

The results of Bartlett’s test of sphericity (χ^2^ = 1355, df = 66, *p* < 0.001) and the Kaiser–Meyer–Olkin test (overall MSA: 0.79, MSA range: 0.69–0.92) indicated that the dataset was suitable for PCA analysis. The 12 variables were positively related, giving a correlation coefficient between 0.10 and 0.99 (Figure 4). PCA was performed on 41 individuals, which were described by 12 variables. PCA transformed the 12 variables into 12 uncorrelated principal components. The first and second components contained 92% of the total variability and eigenvalues of 7.8 and 3.3, respectively. Therefore, the first two components were retained for further analysis. All 12 variables showed a positive correlation (0.54–0.88) with the first component. All the six variables related to the incubation temperature of 15 °C showed a negative correlation (−0.39 to −0.55), and the six variables related to the 25 °C treatment showed a positive correlation (0.51–0.72) to the second component. Most variables within a temperature were positively correlated, while no correlation was observed among the variables between temperatures. The quality of representation was assessed based on the squared coordinates (cos^2^) (Table S1). A cos^2^ value of 1 indicates that the two components perfectly represent the variable. Both the minimum and maximum pollen tube lengths at 15 °C and 25 °C showed lower cos2 values (0.61–0.84) than the remaining variables (>0.95). The contribution was assessed to identify the most significant variables (Table S1). The minimum (7.51%) and maximum (7.75%) pollen tube length at 15 °C, alongside the minimum (5.63%) and maximum (7.03%) pollen tube length at 25 °C, had a total contribution toward the two principal components below the expected average of 8.33%. The remaining eight variables exceeded the average threshold value of 8.33%, and were within the range between 8.78% and 9.18%.

#### 2.2.2. Hierarchical Clustering (HC) and K-Means Clustering

**Figure 5:**
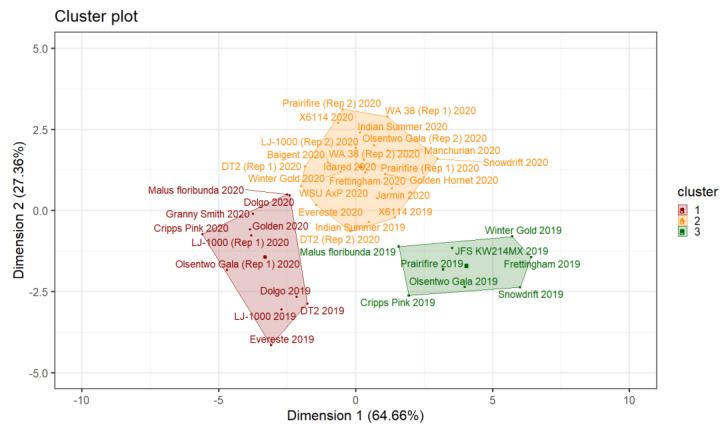
Final partitioning using K-means clustering. Projections are shown on the first and second dimensions, which explains the 92% total variance. Individuals are color-coded based on their cluster membership (cluster 1 = red, cluster 2 = yellow, cluster 3 = green).

Hierarchical clustering was used as an initial step to determine the number of clusters based on the relative loss of inertia. The applied algorithm suggested a three-cluster solution, with 14 individuals in the first, 20 individuals in the second, and 7 individuals in the third cluster. The hierarchical clustering information was used to initialize further partitioning using the K-mean algorithm. Consequently, the cluster memberships for some individuals were rearranged. The first, second, and third clusters comprised 11, 22, and 8 individuals, respectively (Figure 5). Results from the final partitioning showed that individuals in cluster 1 had low coordinates on the first (v.test = −4.55, cluster mean = −3.31, cluster sd = 1.11, overall sd = 2.79, *p* < 0.001) and second (v.test = −3.03, cluster mean = −1.43, cluster sd = 1.49, overall sd = 1.81, *p* = 0.002) dimension. Individuals in the second cluster showed high coordinates on the second dimension (v.test = 5.03, cluster mean = 1.34, cluster sd = 0.98, overall sd = 1.81, *p* < 0.001), and cultivars in cluster 3 had high coordinates on the first (v.test = 4.52, cluster mean = 4.05, cluster sd = 1.72, overall sd = 2.79, *p* < 0.001) and low coordinates on the second (v.test = −2.94, cluster mean = −1.71, cluster sd = 0.64, overall sd = 1.81, *p* = 0.003) dimension. The distance between cultivars and their corresponding cluster centroid was evaluated to determine the most representative cultivar for each cluster. The three most representative cultivars were ID 21 (A) (‘LJ-1000’, Rep 1, 2020), ID 12 (‘Golden’, 2020), and ID 14 (‘Granny Smith’, 2020) for the first cluster, ID 15 (‘Idared’, 2020), ID 11 (‘Frettingham’, 2020), and ID 31 (B) (‘WA 38’, Rep 2, 2020) for the second, and ID 25 (‘Olsentwo Gala’, 2019), ID 19 (‘JFS KW214MX’, 2019), and ID 27 (‘Prairifire’, 2019) for the third cluster.

Table 1 shows the statistical characterization of the three distinguished clusters. Overall, individuals in clusters 1 and 2 have significantly shorter pollen tubes at 15 °C compared to individuals in cluster 3. There was no significant difference between individuals from the first and second clusters at 15 °C. The pollen tube lengths of individuals in the first cluster were significantly shorter at 25 °C compared to individuals in the second and third clusters.

##### Pollen Tube Growth Performance between Years

Thirteen individuals were evaluated in 2019 and 2020 to determine the between-year variability. Only three individuals (‘Dolgo’, ‘Indian Summer’, and ‘X6114’) were in the same cluster during those two consecutive years. Seven individuals, including ‘DT2’, ‘Everest, ‘Frettingham’, ‘LJ-1000’, ‘Prairifire’, ‘Snowdrift’, and ‘Winter Gold’, showed a variable performance. ‘Cripps Pink’, Malus floribunda, and ‘Olsentwo Gala’ showed high between-year variability. All three cultivars showed good performance in 2019 (cluster 3) and low performance in 2020 (cluster 1). Interestingly, the only cultivars that were closest to the third cluster center were ‘Cripps Pink’, ‘Frettingham’, ‘JFS KW214MX’, Malus floribunda, ‘Olsentwo Gala’, ‘Prairifire’, ‘Snowdrift,’ and ‘Winter Gold’ in 2019 (Figure 5). These six cultivars were located closest to the second cluster’s centroid in 2020, indicating a lower overall performance at 15 °C in 2020. The average coordinates of the 13 individuals in 2019 were 1.73 and −1.81 on the first and second dimensions, respectively. In 2020, the average coordinates of these cultivars were −0.82 on the first and 1.18 on the second dimension.

##### Pollen Tube Growth Performance Within a Years

Five individuals, including ‘DT2’, ‘LJ-1000’, ‘Olsentwo Gala’, ‘Prairifire’, and ‘WA 38’, were evaluated at two-time points in 2020 to determine the within-year variability. Three individuals (‘DT2’, ‘Prairifire’, and ‘WA 38’) showed a consistent performance, while the performance of ‘LJ-1000’ and ‘Olsentwo Gala’ varied between sampling times. The first time point of both individuals was assigned to the first cluster, while the second time point was assigned to the second cluster.

#### 2.2.3. Determine Thresholds for Cluster Membership Using a Tree-Based Model

**Figure 6:**
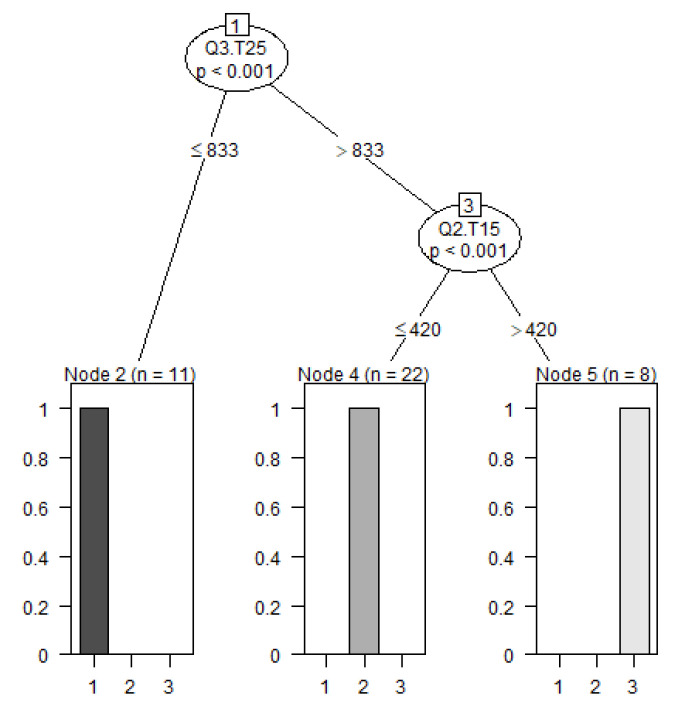
Decision tree model separating 41 cultivars into three categories (clusters) based on the third quartile at 25 °C (Q3.T25) and second quartile at 15 °C (Q2.T15).

A decision tree model was used to predict class membership based on 12 variables (Figure 6). Individuals can be grouped based on two variables: the third quantile at 25 °C (Q3.T25) and the second quantile at 15 °C (Q2.T15). If Q3.T15 is equal to or smaller than 833 µm, the individual belongs to the first cluster. The Q2.T15 value separates the second cluster from the third cluster. If the Q2.T15 value is smaller or equal to 420 µm, then the individual belongs to the second cluster.

### 2.3. Using Predictive Modeling: A Case Study of 29 Apple Accessions

**Figure 7:**
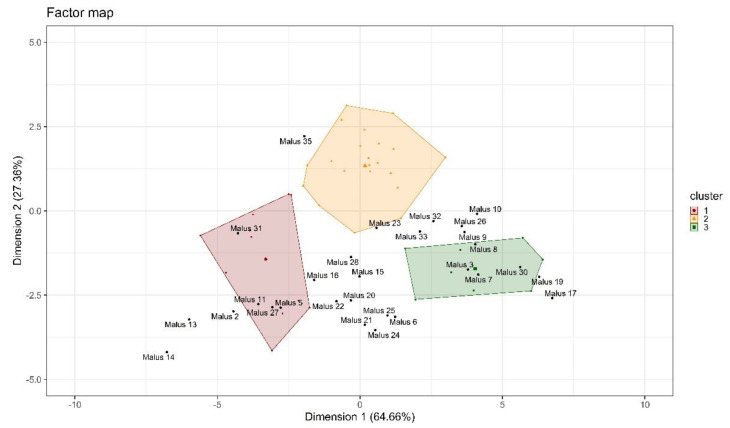
Factorial map of individuals projected according to their position based on 12 variables. Red (cluster 1), yellow (cluster 2), and green (cluster 3) points represent the observations of the initial clustering. Predicted projections of new observations (black) based on the initial PCA.

The coordinates on the factor map of 29 apple accessions were predicted using the results of the subgroups previously identified. The accessions were relatively evenly distributed around the first dimension, with a tendency to lower the second dimension’s coordinates (Figure 7). The accessions were evaluated based on their position on the first and second components, alongside the distance to the nearest cluster center. Twelve accessions (41.4%) were closest to the centroid of the first cluster (Dim 1: −2.86, Dim 2: −1.14), 3 accessions (10.3%) were closest to the second (Dim 1: 0.45, Dim 2: 1.39), and 14 accessions (48.3) were closest to the third cluster (Dim 1: 4.43, Dim 3: −1.69). Several accessions were located outside the initial bounding box of three of their clusters (Figure 7). Accessions closest to the center of the first cluster included Malus 2, Malus 5, Malus 11, Malus 13, Malus 14, Malus 15, Malus 16, Malus 20, Malus 21, Malus 22, Malus 27, and Malus 31, while Malus 23, Malus 28 and Malus 35 were closest to the second cluster center. Accessions closest to the third cluster included Malus 3, Malus 6, Malus 7, Malus 8, Malus 9, Malus 10, Malus 17, Malus 19, Malus 24, Malus 25, Malus 26, Malus 30, Malus 32, and Malus 33. The cluster memberships of the 29 Malus accessions were also determined using thresholds from the decision tree approach. The predicted cluster membership of all accession, except for Malus 15, which was assigned to the first cluster using the PCA approach and to the second cluster using the decision tree approach, was like the PCA approach (Table S2).

## 3. Discussion

### 3.1. Effects of Incubation Time, Germination Media, and Germination Technique on Pollen Tube Growth

Understanding the effect of various environmental conditions on pollen tube growth is a requirement to optimize in vitro screening assays. Several studies have already aimed to optimize certain parameters related to the germination medium or assay [7,31,32,33]. This study aimed to optimize the in vitro protocol for apples by investigating the effects of eight incubation temperatures, seven germination media, and four germination techniques on the pollen tube growth of five domestic apple cultivars. All three factors significantly affected pollen tube growth. Selecting an appropriate incubation time is an important step to ensure the compatibility of the results.

Commonly reported incubation times for apple pollen range from 2 h [12] to 24 h [34] but can be as high as 32 [15] or 72 h [16]. The effect of incubation on pollen germination and pollen tube length follows a linear plateau model. Consequently, the plateau phase must be reached if the research goal is to determine the maximum pollen tube length. However, reaching the plateau phase for pollen tube length can have some disadvantages. First, the pollen suspension can dry out on the agar during the incubation time and affect the pollen tube growth rate. Second, pollen tube lengths can exceed the field of view after a long incubation period. This would consequently result in biased sampling because long tubes that exceeded the field of view would be excluded from the analysis.

Brewbaker and Kwack [35] developed a basal germination media suitable for various plants by investigating the effect of different components on 89 flowering plants belonging to 39 different families and 79 genera. The basic germination medium contained 100 g L^−1^ sucrose, 100 mg L^−1^ boric acid, 300 mg L^−1^ calcium nitrate, 200 mg L^−1^ magnesium sulfate, and 100 mg L^−1^ potassium nitrate in distilled water. Since then, the Brewbaker and Kwack media have become one of the standard germination media for in vitro pollen germination experiments, and several modifications have been made to optimize the medium for specific plant types [36,37,38,39,40].

The different components of the medium have unique functions. For example, sucrose acts as an osmotic agent [41], energy source, and signaling molecules [42], affecting both pollen germination and pollen tube growth. Sucrose follows a biphasic curve; therefore, concentrations below and above the optimum concentrations will negatively affect pollen germination and tube growth. Calzoni et al. [12] recommended the use of 68 g L^−1^ sucrose for optimal germination. However, most current protocols for apple pollen range between 100 g L^−1^ and 150 g L^−1^. Boron is believed to stimulate ATP hydrolysis and H^+^ transport as shown by Obermeyer et al. [43] on ungerminated lily pollen, and several studies have reported that endogenous boric acid promotes pollen germination and tube length in numerous plant species, including almond [44], pineapple [45], apricot [46], apple [46], cherry [46], kiwifruit [47], lychee [48], mango [49], peach [46], pear [46], plum [46], and pomegranate [50].

Specifically for apple pollen, Calzoni et al. [12] showed with their experiments on ‘Golden Delicious’ and ‘Starkrimson’ that relatively low concentrations between 10 and 20 mg L^−1^ are sufficient for maximum pollen germination and that increasing concentrations had a larger effect on pollen tube growth than on pollen germination. The report supports our observation, where the Media 3 with 10 mg L^−1^ did not significantly differ from our standard media, which contained 25 mg L^−1^. In contrast to our work, Calzoni et al. [12], Imani et al. [14], and Mehri et al. [51] investigated the effects of different calcium nitrate concentrations and found a positive effect on pollen germination. Calzoni et al. [12] also reported a positive effect of 200–300 mg L^−1^ calcium nitrate on pollen tube length. A potential reason for this discrepancy could be the pollen population effect. Giulivo and Ramina [52] hypothesized that calcium plays an important role in smaller populations and that large pollen populations contain enough growth factors to compensate for lower calcium levels. However, Giulivo and Ramina’s [52] experiments focused on the pollen germination rate of ‘Golden Delicious’, and the population effect on pollen tube length was not investigated. Brewbaker and Kwack [35] further recommended adding magnesium sulfate and potassium nitrate. However, those two compounds have not been shown to promote pollen germination in apples [14,51]. Calzoni et al. [12] also compared the hanging and sitting drop techniques to suspension culture and showed that the germination rate in drop techniques was inferior. The authors recommended using the suspension culture because of the higher germination rates and the easier handling. The suspension culture was also recommended to overcome problems of varying pollen densities. However, it is unclear why the varying pollen densities represented a problem for the authors because three of their methods included the suspension of pollen in liquid germination media. Furthermore, results regarding the effect of the germination technique on pollen tube growth were not presented.

Based on the present results, pollen suspension combined with 1% agar plates for a standardized protocol is recommended. The main benefit of this method is the ability to standardize the pollen density of the suspension. An incubation period of four hours worked best under our conditions because longer incubation times resulted in pollen tubes that exceeded the maximum field of view at the lowest magnification of our equipment (5× objective, 10× lens) and resulted in biased measurements. Furthermore, the pollen suspension on agar plates might dry out during long incubation times or higher temperatures, which could affect the pollen tube growth rate. Although some differences between the investigated germination media were significant, all medium, except for the control medium (distilled water), seemed suitable for in vitro germination of apple pollen.

### 3.2. Identification and Characterization of Subgroups

In this study, we utilized hierarchical clustering on principal components (HCPC) to analyze the in vitro pollen tube growth performance of 41 individuals at two temperatures. The individuals were grouped into three clusters based on their responses. Cluster 1 individuals showed low performance at 15 °C and 25 °C, while individuals in cluster 2 showed low performance at 15 °C and high performance at 25 °C. Individuals in the third cluster showed high performance at both temperatures. In theory, there is also the possibility of a fourth cluster, which would describe individuals with high performance at 15 °C and low performance at 25 °C. However, this combination was not observed during the two years of testing.

The six variables used for the performance evaluation (minimum, average, maximum, 25th, 50th, and 75th percentiles) within a temperature were grouped together on the variable correlation circle, indicating that they are highly correlated. However, most variables between the two temperatures were orthogonal, indicating that they are independent. Therefore, individuals with low performance at 15 °C do not necessarily have a low performance at 25 °C. Furthermore, two incubation temperatures were required to differentiate the three subgroups.

Studies on almonds [22], coconut [53], cotton [21], and groundnut [20] have shown that cultivars can have different cardinal temperatures for pollen germination and tube growth. The variation in cardinal temperatures could explain some of the cultivar differences. However, cardinal temperatures for in vitro pollen germination rates and tube length have not been determined for different apple cultivars. 

Our results from the clustering of 13 individuals tested in 2019 and 2020 indicated a lower performance in 2020. Overall, six of the individuals that were assigned to the third cluster in 2019 were assigned to the second cluster in 2020. This would indicate that the performance at 15 °C, but not at 25 °C, was reduced. The reason for this variable response is unknown. Earlier studies have already reported year-to-year variation in almonds [54], black wattle [55], chestnuts [56], and pine [57]. In our case, we observed some freezing temperatures a few days prior to the first flower sampling date on 19 April 2020. The last frost dates were 19 March (0.0 °C) and 17 April (−1.7 °C) in 2019 and 2020, respectively.

Both PCA and clustering are appropriate only if specific criteria are fulfilled [58]. We used Bartlett’s test of sphericity and the Kaiser–Meyer–Olkin (KMO) measure of sample adequacy before the PCA to determine the suitability of dimension reduction techniques on our dataset. A significance level below 0.05 for Bartlett’s test and a KMO value above 0.5 indicate that a dataset is suitable for data reduction techniques. Furthermore, the Hopkins statistic was used to determine the clustering tendency of the original (41 individuals and 12 variables) and reduced (41 individuals and 2 dimensions) data sets. The original and reduced datasets had Hopkins scores of 0.70 and 0.55, respectively. Both scores were above the threshold of 0.5. Therefore, it was assumed that the data set contained meaningful clusters. Notably, several indications can be used to determine the clusterability of a dataset. Hopkins statistics were chosen because of their ability to treat outliners as small clusters, as shown by Adolfsson et al. [58], and because of the relatively low dimensionality of our dataset. A further important step is the selection of retained dimensions [59]. In this study, PCA resulted in 12 dimensions, of which only two were retained. The number of retained dimensions was based on the eigenvalues of the components, a visual inspection of the screen plot, and the cumulative explained variance. Recall that none of the methods are based on an actual test statistic. In summary, the proposed classification of 41 apple individuals based on their in vitro pollen tube performance may help further studies to classify cultivars based on the reference set provided by this study.

### 3.3. Using Predictive Modeling: A Case Studies of 29 Apple Accessions

Using predictive projection based on the PCA of the above-described reference set has some limitations. First, cluster memberships should only be assigned to new individuals if the same in vitro germination protocol was used. The use of different germination conditions will result in unreliable projections and decrease the reliability of the results. Second, the initial projection of the reference set covers a certain range on the plane. The range of coordination from the initial projection ranged from −5.45 to 8.30 on the first and from −3.77 to 2.72 on the second dimension. Assigning a class membership based on the distance between new individuals and the closest cluster center is reasonable only if those individuals are near the boundary box of the reference set, as extrapolations can induce greater uncertainty. Using the two rules from the decision tree model has also been shown to be an accurate method of determining the cluster membership for new individuals. Last, the described approach characterized the accession based on their pollen tube performance without any maternal effects, such as stigmatic receptivity or biochemical or biophysical properties of the transmitting tissue. Significant interactions between paternal and maternal parents have been shown to affect pollen tube growth in apples [60]. Therefore, while in vitro-based screenings can help to identify potential advanced cultivars within a larger population, in vivo trials are necessary to determine the effectiveness of a pollen source for a specific cultivar of interest.

## 4. Materials and Methods

### 4.1. Effects of Incubation Time, Germination Media, and Techniques on Pollen Tube Growth

Frozen pollen from five apple cultivars (‘Delicious’, ‘Gala’, ‘Granny Smith’, ‘Honeycrisp’, and ‘Rome Beauty’) was acquired from a commercial company (Firman Pollen Co. Inc., Yakima, WA, USA) in December 2016 and stored at −20 °C upon arrival. The pollen samples were used to investigate the effect of incubation time, germination technique, and germination medium on pollen tube length. Prior to each experiment, a subsample of frozen pollen was transferred to a 1.5 mL micro-centrifuge tube. Subsequently, the pollen was acclimated by placing the tube into a closed glass vial that contained moist filter paper (Whatman #1) on the bottom. Samples were acclimated at 25 °C for 60 min.

#### 4.1.1. Effect of Incubation Time

One mL of germination medium 7 (see Table 2 for media composition) was added to the tubes, and samples were gently vortexed for 3–5 s to suspend the pollen. Afterward, 200 µL of pollen suspension was transferred to 1% agar plates. The samples were dark-incubated at 25 °C for 1, 2, 4, 6, 8, 10, 12, and 24 h.

#### 4.1.2. Effect of Germination Technique

Four germination techniques, namely (1) agar plate in combination with pollen dusting, (2) agar plate in combination with pollen suspension, (3) hanging drop slide, and (4) sitting drop slide, were compared to determine the effect on pollen tube growth. Agar plates, for the first technique, were made by autoclaving 100 g L^−1^ sucrose, 25 mg L^−1^ boric acid, and 10 g L^−1^ agar in distilled water for 60 min at 105 °C. Plates were poured after the medium cooled down to approximately 65 °C, dried for 6 h under a sterile laminar flow hood, and stored at 4 °C until further use. All plates were acclimated at 25 °C for 1 h before each experiment. Acclimated pollen was transferred to a 100 µL cell strainer and sprinkled over the agar plates using a fine brush. A liquid germination medium containing 100 g L^−1^ sucrose and 25 mg L^−1^ boric acid was used for the germination techniques that utilized a pollen suspension (techniques 2–4). The agar plate methodology combined with pollen suspension was like the one used for the incubation time. As for the hanging and sitting drops, three O-rings were evenly spaced onto a microscopy slide. Afterward, one drop (20 µL) of the pollen suspension was pipetted into each O-ring center. A second microscopy slide was placed on top of the O-rings, and two binder clips were used to seal the two slides. The difference between the hanging and sitting drop techniques was the orientation of the drops. Slides for the sitting drop techniques were left in the original position, while slides for the hanging drop were flipped so that the drops faced downward. The amount of pollen on the hanging and sitting drop slides were low because of the reduced volume. Therefore, each slide comprising of three drops was considered a replication. All samples were dark-incubated at 25 °C for 2 h.

#### 4.1.3. Effect of Germination Media

Five germination media, adding to the control (distilled water) and a standard germination medium, were used (Table 2). These were compared to evaluate the influence of each on the pollen tube length. One mL of every medium was added to the sample tube. Tubes were vortexed for 3–5 s to promote dispersion, and 200 µL of the pollen suspension was transferred onto a 1% agar plate. All plates were dark-incubated at 25 °C for 2 h.

### 4.2. Identification and Characterization of Subgroups

Unopened flowers from 14 apple individuals (‘Cripps Pink’, ‘Dolgo’, ‘DT2’, ‘Evereste’, ‘Frettingham’, ‘Indian Summer’, ‘Ivory Spear’, ‘LJ-1000’, Malus floribunda, ‘Olsentwo Gala’, ‘Prairifire’, ‘Snowdrift’, ‘Winter Gold’, and ‘X6114’) were harvested at the late balloon stage between April and May in 2019. Furthermore, flowers from 20 individuals (‘Baigent’, ‘Cripps Pink’, ‘Dolgo’, ‘DT2’, ‘Evereste’, ‘Frettingham’, ‘Golden Delicious’, ‘Golden Hornet’, ‘Granny Smith’, ‘LJ-1000’, ‘Idared’, ‘Indian Summer’, ‘Ivory Spear’, Malus floribunda, ‘Manchurian’, ‘Marilee’, ‘Olsentwo Gala’, ‘Prarifire’, ‘Snowdrift’, ‘WA 38’, ‘WSU AxP’, and ‘X6114’) were harvested between April and May 2020. The 2020 dataset includes all individuals, except for ‘Ivory Spear’, which was tested in 2019. Furthermore, flowers from ‘DT2’, ‘LJ-1000’, ‘Olsentwo Gala’, ‘Prairifire’, and ‘WA 38’ were harvested at two different time points in 2020. The different time points were labeled as “Rep 1” and “Rep 2” and every cultivar, year, and rep combination was treated as a separate individual (41 in total). All accessions, except for ‘Ivory Spear’, Malus floribunda, ‘Puget Spice’, and ‘Winter Gold’, which were grown in pots at the Tree Fruit Research and Extension Center in Wenatchee (WA, USA), were planted at the WSU Sunrise Research Orchard (Rock Island, WA, USA). The apple collection was planted between 2007 and 2018.

Anthers from 10 flowers were separated using fine tweezers, collected in 5.0 mL Eppendorf tubes, and air-dried for 24 h in a drying chamber (temperature: 25.0 ± 1.0 °C, rel. humidity: 24.4 ± 5.3%). Afterward, 2.5 mL of liquid germination media (100 g L^−1^ sucrose and 25 mg L^−1^ boric acid in deionized water, observed pH = 6.1 ± 0.2) was added to the sample tubes. Tubes were gently vortexed for 3–5 s to promote pollen release, and 200 µL of the pollen suspension was transferred onto a 1% agar plate. Petri-dishes were incubated for 4 h at 15 °C and 25 °C.

### 4.3. Using Predictive Modeling: A Case Study of 29 Apple Accessions

A case study was performed to demonstrate the implementation and utilization of the identified and characterized subgroups using the HCPC/decision tree modeling approach. Flowers from 29 unreleased apple accessions were harvested, dried, germinated, and analyzed using the methods described above (see Section 4.2). The accessions were part of a crabapple screening program and were planted in 2017 at the WSU Sunrise Research Orchard (Rock Island, WA, USA) after being held in 40 L containers for up to two years. All trees were on ‘Dolgo’ rootstocks. Samples were collected and analyzed between April and May 2019.

### 4.4. Microscopic Observation and Pollen Tube Measurements

All samples were evaluated under an inverted microscope (Leica DMi1, Leica Microsystems, Germany). Between 6 and 10 images from different fields of view were captured from each of the four replications using a Canon Rebel T6 DSLR camera. Ideally, the five longest pollen tube lengths from five different images (field of views) were estimated for every replication using ImageJ software (Schneider et al., 2012). A microscope calibration slide (MR095, AmScope, Irvine, CA, USA) was used to spatially calibrate the images in ImageJ to a 1 µm resolution. In addition to the pollen tube length, measurements of the incubation time experiment and the pollen tube growth rate (µm h^−1^) for each observation were calculated by dividing the pollen tube length by the incubation time.

### 4.5. Statistical Analysis

Statistical analyses were performed using R 4.0.3 [62]. R packages and functions are reported as ‘package name:function name’. Analysis of variance was performed to analyze the effect of incubation time, germination media, and germination technique on pollen tube length. Diagnostic plots were used to check linearity (residual vs. fitted plot), normality (normal Q-Q plot), homoscedasticity (scale location plot), and influential observations (residual vs. leverage plot). Multiple comparisons were either performed using Fisher’s least significant difference test using agricolae:LSD.test (version 1.3.3) [63], if the sample size and variance were equal, or the Games-Howell test using userfriendlyscience:oneway (version 0.7.2) [64] combined with multcompView:multcompLetters (version 0.1.8) [65], if the sample size and variances were unequal. A six-number summary statistic was calculated for the second (see 4.2) and third (see 4.3) datasets using plyr:ddply (version 1.8.6) [66]. The summary statistic was based on 100 observations (25 observations per replication) for every individual and temperature combination and included the minimum, average, maximum, 25th (Q1), 50th (Q2), and 75th (Q3) percentiles (Tables S2 and S3). PCA was performed on 12 variables using FactoMineR:PCA (version 2.4) [67]. Bartlett’s test of sphericity and the Kaiser–Meyer–Olkin test of sampling adequacy were run before PCA analysis. Test statistics were calculated using psych:cortest.bartlett for Bartlett’s test and psych:KMO for the Kaiser–Meyer–Olkin test (version 2.0.12) [68]. Hierarchical clustering of principal components was performed using FactoMineR:HCPC (version 2.4 [67]. Clustering was based on the Ward criteria for the first two principal components. Cluster tendency was assessed using factoextra:get_cluster_tendency (version 1.0.7) [69]. A decision tree was created using party:ctree (version 1.3.7) [70]. All plots were created using factoextra:fviz_dend, factoextra:fviz_cluster (version 1.0.7) [69], ggplot2:ggplot (version 3.3.2) [71], and ggpubr:ggarrange (version 0.40) [72].

## 5. Conclusions

In this study, new metrics to characterize apple cultivars based on their in vitro pollen tube performance using hierarchical clustering on principal components were described. In total, three distinct clusters were identified and described based on 41 individuals. The resulting reference dataset will allow further studies to add any number of individuals and determine their class membership by predicting their projection on the factor map. Furthermore, cluster membership determination is also possible based on the median (Q2) at 15 °C and third quartile (Q3) at 25 °C, as determined by the decision tree model. However, while the decision tree approach is easier to use, the PCA approach has a better visualization feature. Both PCA and the decision tree approach produce similar results, as shown in this case study with 29 crabapple accessions. The optimized in vitro germination protocol represents a prerequisite to adding new projections to the apple reference set.

## Figures and Tables

**Table 1:** Statistical characterization of clusters identified by hierarchical clustering on principal components.

Temperature (°C)	Variable	Pollen Tube Length (µm)									F _(2, 38)_	*p*
		Cluster 1 (*n* = 11)			Cluster 2 (*n* = 22)			Cluster 3 (*n* = 8)				
		Mean	95% CI	Group	Mean	95% CI	Group	Mean	95% CI	Group		
15	Minimum	119	97, 141	b	145	129, 160	b	289	263, 315	a	59.4	<0.001
	Average	283	242, 325	b	326	297, 356	b	589	540, 638	a	54.5	<0.001
	Maximum	544	445, 642	b	567	497, 637	b	968	853, 1084	a	20.8	<0.001
	Q1 ^(1)^	218	182, 254	b	267	241, 292	b	487	445, 529	a	53.7	<0.001
	Q2 ^(2)^	273	233, 314	b	318	290, 347	b	577	530, 624	a	56.8	<0.001
	Q3 ^(3)^	334	286, 383	b	381	346, 415	b	681	623, 738	a	51.5	<0.001
25	Minimum	339	284, 395	b	545	506, 584	a	490	425, 455	a	18.8	<0.001
	Average	619	578, 660	b	879	850, 908	a	902	854, 950	a	63.3	<0.001
	Maximum	1002	911, 1094	b	1264	1199, 1329	a	1399	1292, 1506	a	18.3	<0.001
	Q1 ^(1)^	531	485, 576	b	788	756, 821	a	778	725, 832	a	47.0	<0.001
	Q2 ^(2)^	611	569, 654	b	874	843, 904	a	895	845, 945	a	59.5	<0.001
	Q3 ^(3)^	697	657, 738	b	969	964, 1059	a	1012	964, 1059	a	73.6	<0.001

Note: Means followed by the same letter in the row do not significantly differ by Tukey test (α = 0.05). ^(1)^ first quartile, ^(2)^ second quartile, ^(3)^ third quartile.

**Table 2:** Composition of in vitro pollen germination media.

Media ID	Sucrose (g L^−1^)	Boric Acid (mg L^−1^)	Calcium Nitrate (mg L^−1^)	Reference
Medium 1	0	0	0	-
Medium 2	68	20	300	[12]
Medium 3	100	10	0	[60]
Medium 4	150	100	300	[14]
Medium 5	125	22.5	250	[61] ^1^
Medium 6	150	200	300	[17]
Medium 7	100	25	0	Roeder et al. (2021)—current study

^1^ Values were reported as range. Average was used for this study.

## Data Availability

All the data are contained within the article or available online as supplementary materials (Tables S1–S3).

## Supplementary Materials

The following are available online at https://www.mdpi.com/article/10.3390/plants10071460/s1, Table S1: Characterization of variables (minimum, average, maximum, Q1, Q2, and Q3) based on their correlation, quality of representation and contribution to the first and second dimension at 15 °C and 25 °C, Table S2: Pollen tube length related variables (minimum, average, maximum, first, second, and third quartile for 41 individuals incubated at 15 °C and 25 °C, Table S3: Pollen tube length related variables (minimum, average, maximum, first, second, and third quartile for 29 individuals incubated at 15 °C and 25 °C.
