# Supplementary material for: Novel Metrics to Characterize In Vitro Pollen Tube Growth Performance of Apple Cultivars
Source: Plants (Basel). 2021 Jul 16;10(7):1460. doi: 10.3390/plants10071460 (PMC8309383; doi:10.3390/plants10071460)
Supplement: Supplementary file 1 [file plants-10-01460-s001.zip › plants-1296942-supplementary.pdf]

plants

MDPI

Article

Novel Metrics to Characterize *In Vitro* Pollen Tube Growth Performance of Apple Cultivars

Stefan Roeder [1,2], Sara Serra [1,2] and Stefano Musacchi [1,2,*]

Supplementary Materials

Table S1. Characterization of variables (minimum, average, maximum, Q1, Q2, and Q3) based on their correlation, quality of representation and contribution to the first and second dimension at 15 °C and 25 °C.

Incubation temperature	Variable	Correlation		Representation		Total	Contribution (%)		Total contri-bution (%)
		Dim. 1	Dim. 2	Dim. 1	Dim. 2	representation	Dim. 1	Dim. 2	
15 °C	minimum	0.83	-0.35	0.69	0.12	0.81	8.9	4.0	7.5
	average	0.88	-0.47	0.77	0.22	0.99	9.9	7.4	9.2
	maximum	0.75	-0.53	0.56	0.28	0.84	7.2	9.2	7.8
	first quartile (Q1)	0.88	-0.44	0.77	0.2	0.97	9.9	6.5	9.0
	second quartile (Q2)	0.88	-0.46	0.77	0.21	0.99	9.9	7.1	9.1
	third quartile (Q3)	0.87	-0.48	0.76	0.23	0.99	9.7	7.6	9.1
25 °C	minimum	0.55	0.56	0.3	0.31	0.61	3.9	10.2	5.6
	average	0.8	0.58	0.64	0.34	0.98	8.2	11.2	9.1
	maximum	0.85	0.2	0.72	0.04	0.76	9.3	1.3	7.0
	first quartile (Q1)	0.71	0.67	0.5	0.45	0.95	6.4	15.0	8.8
	second quartile (Q2)	0.79	0.59	0.62	0.35	0.97	7.9	11.6	9.0
	third quartile (Q3)	0.83	0.52	0.69	0.27	0.96	8.9	9.0	8.9

Table S2. Pollen tube length related variables (minimum, average, maximum, first, second, and third quartile for 41 individuals incubated at 15 °C and 25 °C.

ID	Individual	Pollen tube length												Cluster membership (HCPC)
		15 °C						25 °C						
		Min	Avg	Max	Q1	Q2	Q3	Min	Avg	Max	Q1	Q2	Q3	
1	Baigent 2020	120	272	483	230	272	311	501	847	1136	738	856	959	2
2	Cripps Pink 2019	273	534	853	448	516	628	418	773	1266	631	763	897	3
3	Cripps Pink 2020	101	164	283	122	156	190	277	572	795	508	572	640	1
4	Dolgo 2019	105	351	835	231	344	452	272	601	1389	468	565	711	1
5	Dolgo 2020	133	252	430	193	242	296	482	727	1104	656	728	779	1
7 (A)	DT2 (Rep 1) 2020	129	248	405	198	243	292	535	796	1053	716	802	865	2
7 (B)	DT2 (Rep 2) 2020	230	378	511	351	386	415	441	783	1068	704	778	864	2
6	DT2 2019	119	412	792	315	415	485	354	609	1056	504	604	691	1
8	Evereste 2019	126	387	874	305	362	461	239	509	957	409	501	583	1
9	Evereste 2020	125	305	532	262	301	350	450	765	1102	675	756	868	2
10	Frettingham 2019	254	700	1164	583	674	813	637	983	1638	872	972	1066	3
11	Frettingham 2020	150	335	495	302	338	381	399	917	1254	824	904	1016	2
12	Golden 2020	121	220	394	172	210	258	268	657	1002	591	643	722	1
13	Golden Hornet 2020	150	378	593	320	364	438	516	909	1333	823	908	1006	2
14	Granny Smith 2020	104	215	376	172	212	255	421	649	1050	556	629	733	1
15	Idared 2020	180	322	572	264	316	375	556	885	1230	804	880	974	2
16	Indian Summer 2019	200	376	654	308	358	438	438	810	1489	678	770	937	2
17	Indian Summer 2020	120	304	496	250	295	364	663	919	1151	848	918	988	2
18	Jarmin 2020	183	395	598	321	396	465	499	904	1373	787	899	1002	2
19	JFS KW214MX 2019	306	562	786	476	558	643	561	901	1228	806	892	986	3
21 (A)	LJ-1000 (Rep 1) 2020	124	255	420	198	246	292	406	625	796	577	620	689	1
21 (B)	LJ-1000 (Rep 2) 2020	107	314	543	239	318	389	640	891	1169	786	890	987	2
20	LJ-1000 2019	170	361	653	306	349	414	295	578	962	434	582	667	1
22	Malus floribunda 2019	227	446	823	358	443	526	331	863	1339	734	851	976	3
23	Malus floribunda 2020	105	244	432	194	238	282	417	729	1165	642	729	833	1
24	Manchurian 2020	172	347	549	295	346	389	666	910	1449	826	902	979	2
26 (A)	Olsentwo Gala (Rep 1) 2020	102	257	490	193	234	294	300	552	750	494	550	622	1
26 (B)	Olsentwo Gala (Rep 2) 2020	144	328	501	271	324	382	621	911	1361	820	915	993	2
25	Olsentwo Gala 2019	307	604	1010	507	593	704	476	865	1368	753	852	976	3
28 (A)	Prairifire (Rep 1) 2020	127	340	739	251	324	411	537	915	1227	833	918	997	2
28 (B)	Prairifire (Rep 2) 2020	102	230	504	163	211	277	611	932	1304	842	928	1019	2
27	Prairifire 2019	234	566	986	450	554	656	395	886	1317	751	894	1004	3
29	Snowdrift 2019	340	676	1228	554	655	753	482	954	1624	793	930	1089	3
30	Snowdrift 2020	213	432	648	370	420	490	602	1002	1485	921	983	1084	2
31 (A)	WA 38 (Rep 1) 2020	127	313	594	260	314	358	676	989	1283	891	982	1078	2
31 (B)	WA 38 (Rep 2) 2020	128	334	638	236	322	406	656	869	1286	793	862	942	2
32	Winter Gold 2019	370	623	897	520	624	722	623	993	1410	885	1006	1098	3
33	Winter Gold 2020	106	310	454	266	308	352	492	835	1362	769	828	899	2
34	WSU AxP 2020	123	254	462	204	246	294	463	767	1155	674	744	868	2
35	X6114 2019	128	423	1052	312	370	512	425	888	1323	746	894	1021	2
36	X6114 2020	121	243	451	191	234	283	600	901	1216	845	902	972	2

Table S3. Pollen tube length related variables (minimum, average, maximum, first, second, and third quartile for 29 individuals incubated at 15 °C and 25 °C.

Accession	Pollen tube length												Cluster membership	
	15 °C						25 °C							
	Min	Avg	Max	Q1	Q2	Q3	Min	Avg	Max	Q1	Q2	Q3	HCPC	DT
Malus 2	119	300	558	251	280	341	276	497	874	431	490	553	1	1
Malus 3	237	580	923	506	559	658	446	874	1657	761	864	970	3	3
Malus 5	156	366	700	280	347	437	316	583	837	518	579	637	1	1
Malus 6	292	518	869	453	515	573	348	738	1058	632	740	840	3	3
Malus 7	276	601	990	528	615	665	510	888	1372	771	886	1011	3	3
Malus 8	314	561	927	475	578	626	643	917	1290	809	901	1002	3	3
Malus 9	256	533	853	442	508	602	470	942	1556	801	946	1035	3	3
Malus 10	258	514	994	429	499	590	600	964	1549	818	957	1079	3	3
Malus 11	61	312	783	250	305	380	286	549	1090	449	523	610	1	1
Malus 13	112	231	494	178	217	273	177	435	773	372	437	498	1	1
Malus 14	50	247	639	186	241	296	174	362	638	289	344	432	1	1
Malus 15	228	438	767	337	426	526	468	716	1055	637	717	780	2	1
Malus 16	57	407	635	352	406	476	325	671	1141	547	646	767	1	1
Malus 17	392	708	1370	586	658	806	505	980	1451	875	966	1092	3	3
Malus 19	320	686	1197	542	661	787	586	958	1743	813	925	1061	3	3
Malus 20	189	445	923	340	432	524	422	680	1120	570	648	764	1	1
Malus 21	211	501	856	384	493	602	327	676	1108	531	676	810	1	1
Malus 22	64	451	838	344	427	546	242	678	1324	559	658	778	1	1
Malus 23	69	415	860	316	414	512	399	833	1368	687	829	954	2	2
Malus 24	150	491	1053	361	462	612	67	726	1442	592	717	871	3	3
Malus 25	93	547	1024	423	566	648	296	746	1152	602	734	884	3	3
Malus 26	298	521	761	462	523	579	560	939	1356	806	942	1028	3	3
Malus 27	143	349	637	289	343	401	313	552	939	488	563	614	1	1
Malus 28	167	400	721	288	391	487	360	752	1264	628	725	867	2	2
Malus 30	364	640	1072	538	637	722	520	976	1368	885	950	1088	3	3
Malus 31	103	212	458	164	199	241	329	622	892	542	631	703	1	1
Malus 32	273	452	849	389	433	497	515	898	1442	771	882	1027	3	3
Malus 33	111	502	1070	389	477	573	593	862	1262	747	849	969	3	3
Malus 35	66	204	474	137	194	248	516	835	1238	743	833	920	2	2
